# The Effect of Weight Loss in Obese Patients with Chronic Stable Plaque-Type Psoriasis

**DOI:** 10.1155/2013/795932

**Published:** 2013-09-18

**Authors:** Wanjarus Roongpisuthipong, Marinya Pongpudpunth, Chulaporn Roongpisuthipong, Natta Rajatanavin

**Affiliations:** ^1^Division of Dermatology, Department of Medicine, Faculty of Medicine, Ramathibodi Hospital, Mahidol University, Bangkok 10400, Thailand; ^2^Department of Medicine, Faculty of Medicine Vajira Hospital, University of Bangkok Metropolis, Bangkok 10300, Thailand; ^3^Division of Nutrition and Biochemical Medicine, Department of Medicine, Faculty of Medicine, Ramathibodi Hospital, Mahidol University, Bangkok 10400, Thailand

## Abstract

*Background*. Chronic plaque psoriasis is frequently associated with obesity. The effect of a low-calorie diet on psoriasis has not been investigated. *Objective*. The objective was to investigate whether moderate weight loss increases the therapeutic response to topical treatment in obese patients with chronic stable plaque-type psoriasis. *Material and Method*. A 24-week clinical trial was conducted in 10 patients. The efficacy of a low-calorie diet with topical treatment was compared with baseline in obese patients with chronic stable plaque-type psoriasis. The primary measure of clinical response was the Psoriasis Area and Severity Index at weeks 12 and 24. *Results*. At week 12, the mean reduction in body weight was 9.6 percent. There was an improvement from baseline of 50 percent or more in the Psoriasis Area and Severity Index in 50 percent of the patients. The responses as measured by improvements in the Psoriasis Area and Severity Index were paralleled by improvements in global assessments by the physician and the patients and in the Dermatology Life Quality Index. *Conclusion*. Obese patients with chronic stable plaque-type psoriasis increase their response to a low-calorie diet. Lifestyle modifications, including a low-calorie diet, may supplement the pharmacologic treatment of obese psoriasis patients.

## 1. Introduction

Chronic plaque-type psoriasis is a chronic inflammatory skin disorder that affects approximately 2 percent of the world's population and is associated with obesity in 13–34% of cases [1, 2]. The relative risk of psoriasis has been reported to be directly related to body mass index (BMI), and positive correlation between psoriasis severity and BMI has been established [3]. Moreover, epidemiological studies have reported associations between psoriasis and the metabolic syndrome in a dose-response manner. Worldwide, the incidence of obesity and metabolic syndrome has increased dramatically in recent decades [4, 5]. Obesity and metabolic syndrome are a cluster of risk factors including dyslipidemia, hypertension, and insulin resistance and are strong predictors for cardiovascular disease, diabetes mellitus, and stroke [6, 7]. Indeed, psoriasis has also been reported to be an independent risk factor for myocardial infarction, especially in young patients [8]. Psoriasis and obesity share similar mediators of inflammation, mainly tumor necrosis factor-*α* (TNF-*α*) and interleukin-6 (IL-6). The effectors of adipocytic and psoriatic inflammation, mainly adipocytes and macrophages, are derived from a common mesothelial origin [9]. Furthermore, obesity-associated inflammation is characterized by decreases in anti-inflammatory adipokines especially adiponectin [10]. Weight loss in obese patients is associated with decreases in the serum concentrations of inflammatory mediators, including TNF-*α*, IL-6, C-reactive protein, and fibrinogen, and with a concomitant increase in adiponectin which exerts anti-inflammatory and insulin-sensitizing effects [11]. The objective of this study was to investigate whether moderate weight loss (5–10% of body weight) increases the therapeutic response to topical treatment in obese patients with chronic stable plaque-type psoriasis.

## 2. Material and Method

### 2.1. Subjects

Sample size was calculated by using two-test proportion. All subjects were recruited from the dermatology outpatient clinic of Ramathibodi Hospital, Mahidol University. Inclusion criteria for subjects were age more than 18 years, BMI ≥ 30 kg/m^2^, metabolic syndrome, and clinically stable plaque-type psoriasis. Patients with any change in systemic treatment of psoriasis for at least 3 months before enrollment were not included in the study. Patients were permitted to use low or moderate potency of topical corticosteroids during the study. Exclusion criteria were other types of psoriasis (guttate, erythrodermic, and pustular psoriasis), renal and liver impairment, previous or active malignancies, and severe infection. Pregnant or lactating women were also excluded. After signed informed consent, all subjects were visited by one nutrition specialist physician and one dietitian who recorded age, gender, blood pressure, weight, height, BMI, and waist and hip circumference at baseline, 4th, 8th, 12th, and 24th weeks. Two dermatologists recorded age of psoriasis onset, type, and severity of psoriasis. Severity of psoriasis was assessed according to Psoriasis Area and Severity Index (PASI) [12] and Physician's Global Assessment (MDGA) [13] at baseline, 4th, 8th, 12th, and 24th weeks. The proportion of patients with an improvement in the index of at least 50 percent was also determined. Four patient-reported outcomes were assessed: the Dermatology Life Quality Index (DLQI) [14], the Patient's Global Assessment [15],rash, and overall treatment satisfaction scores at baseline, 4th, 8th, 12th, and 24th weeks [16]. Patients were asked to complete a Thai translation of the DLQI which is a self-reported questionnaire to measure how much skin problem has affected the everyday life of patient over the previous 7 days. The biochemical tests including blood sugar, lipid profile, C-reactive protein, erythrocyte sedimentation rate, renal and liver function tests, and hematological parameters were measured at baseline, 12th, and 24th weeks.

The study was reviewed and approved by the Committee on Human Research of Ramathibodi Hospital, Mahidol University. The subjects were informed in detail about the study, and informed consents were obtained from all of them.

### 2.2. Anthropometric Measurement and Body Composition

 The height of each subject was measured using a stadiometer to the nearest 0.1 cm. Body weight was measured using a digital weighing scale (Soehnle 7755, Germany). Body mass and body composition (InBody 720, Germany) were measured in the morning after an overnight fast. Body mass was recorded to the nearest 100 g on a calibrated digital scale with subjects wearing only underwear. The same technician who was unaware of the study details performed analyses.

### 2.3. Statistical Analysis

Baseline characteristics, blood chemistries, PASI score, DLQI, MDGA, and PGA of all subjects were reported by using mean ± standard deviation (SD). Statistical analysis was conducted using SPSS software version 13.0 for windows. All outcome measurements between baseline and each period data were assessed using nonparametric test (Wilcoxon signed-rank test). *P* value < 0.05 was considered statistically significant.

## 3. Results

 Ten obese subjects with chronic stable plaque-type psoriasis were enrolled in the study. The subjects in this study were predominantly male (70 percent), and the mean age was 48.2 years. The mean duration of psoriasis was 15.5 years. Mean weight was 95.9 kg, and BMI was 35.2 kg/m^2^. The mean baseline PASI was 5.72, and DLQI score was 8.00 as shown in Table 1. After receiving very low-calorie diet, the mean reduction in weight was 9.69 percent, and fat mass was 17.9 percent at week 12 as shown in Table 2. At week 12, the PASI 50 response was achieved by 5 patients (50 percent), and the mean percentage improvement in the PASI from baseline was 30.9 percent (*P* < 0.05). The responses as measured by improvements in the Psoriasis Area and Severity Index were paralleled by improvements in global assessments by physicians and patients and in the Dermatology Life Quality Index as shown in Table 3.From week 12 to week 24, there was mega flood crisis in Thailand, the biggest in the past 50 years, which made all subjects stressful and did not adhere to the protocol diet. At week 24, all parameters including Psoriasis Area and Severity Index, Dermatology Life Quality Index, and Global Assessments by Physicians and Patients were more worsening than week 12. Triglyceride, LDL cholesterol, and LDL to HDL cholesterol ratio were significantly decreased from baseline at week 12. There were no significant differences from baseline in fasting blood sugar, total cholesterol, HDL cholesterol, hs-CRP, and ESR as shown in Table 4.

## 4. Discussion 

 The results of this study showed that weight reduction in obese patients with chronic stable plaque-type psoriasis improves their lesions and quality of life. The improvement from baseline in the response as measured by overall treatment and rash satisfaction was statistically significant as early as week 4. At week 12, all parameters including Psoriasis Area and Severity Index, Dermatology Life Quality Index, and Global Assessments by Physicians and Patients were improved. In addition, weight reduction led to a significant decrease in triglyceride, LDL cholesterol, and LDL to HDL cholesterol ratio, a major determinant of CVD risk. There were 2 case reports that demonstrated the complete resolution of psoriasis after significant weight loss in 2 individuals who underwent gastric bypass surgery for obesity [17, 18]. Moreover, there was a randomized, controlled trial that revealed 5 to 10 percent weight loss rendering obese patients with chronic plaque psoriasis responsive to low dose of cyclosporine [19].

 This study had some limitations, such as the relatively small number of patients, physicians who measured results were not blinded, and the mega flood crisis during the study. Further larger studies such as randomized controlled trial would be helpful for this purpose. Although PASI was dropped during the disaster, PASI was not worse than baseline. This effect may be explained by legacy effect of weight reduction; however, this needs further investigations. 

 In conclusion, this study suggests that weight reduction by low-calorie diet in obese patients with chronic stable plaque-type psoriasis increases their response to psoriasis treatment. In addition, quality of life, triglyceride, and LDL cholesterol were improved. Lifestyle modifications, including a low-calorie diet, may support the pharmacologic treatment of obese psoriasis patients.

## Figures and Tables

**Table 1:** Baseline characteristics of patients.

Characteristic	Value
Gender male : female	7 : 3
Mean age, year (SD)	48.2 (7.6)
Mean duration of psoriasis, year (SD)	15.5 (9.1)
Mean BMI, kg/m^2^ (SD)	35.2 (6.3)
Mean body weight, kg (SD)	95.9 (23.6)
Mean waist, cm (SD)	112.3 (14.8)
Mean hip, cm (SD)	115.2 (9.5)
Mean fat mass, kg (SD)	38.6 (14.1)
Mean skeletal mass, kg (SD)	30.9 (6.91)
Mean systolic blood pressure, mmHg (SD)	130.3 (15.8)
Mean diastolic blood pressure, mmHg (SD)	81.8 (8.8)
Mean Psoriasis Area and Severity Index (SD)	5.72 (3.8)
Mean patient assessed by the physician as having moderate or high severity^†^, %	60
Mean patient with self-assessed moderate or high severity^‡^, %	100
Mean overall treatment satisfaction score (SD)	1.60 (2.0)
Mean rash satisfaction score (SD)	1.60 (2.0)
Mean Dermatology Life Quality Index* (SD)	8.00 (4.0)

^†^Data are for patients with a baseline score of 3 (moderate), 4 (severe) or 5 (very severe) on the Physician's Static Global Assessment; scores range from 0 to 5, with 0 indicating no evidence of plaque elevation, erythema, or scaling and 5 indicating severe induration, erythema, and scaling.

^‡^Data are for patients with a psoriasis score of 3 to 5 on the Patient's Global Assessment of Psoriasis; scores range from 0 to 5, with 0 indicating good and 5 indicating severe.

*The Dermatology Life Quality Index ranges from 0 to 30, with 0 indicating best and 30 indicating worst.

**Table 2:** Changes in weight, BMI, and body compositions at weeks 4, 8, 12, and 24.

	Week 4	Week 8	Week 12	Week 24
Mean BMI, kg/m^2^ (SD)	33.2 (5.8)**	32.2 (5.5)**	31.8 (5.6)**	31.2 (5.3)**
Mean body weight, kg (SD)	90.1 (21.6)**	87.5 (20.7)**	86.4 (20.9)**	86.2 (20.1)**
Percentage reduction in body weight	5.9 (1.8)**	8.5 (1.9)**	9.6 (2.6)**	9.8 (2.6)**
Mean fat mass, kg (SD)	35.3 (13.2)**	32.9 (11.8)**	31.8 (12.5)**	31.2 (12.1)**
Percentage reduction in fat mass	8.87 (4.6)**	14.5 (3.6)**	17.9 (6.9)**	19.4 (7.7)**
Mean waist, cm (SD)	109.9 (14.5)*	107.3 (13.5)**	104.2 (13.1)**	103.0 (13.3)**
Mean hip, cm (SD)	112.8 (10.6)*	112.3 (9.5)**	109.9 (7.88)**	108.0 (8.6)**

*P < 0.05 compared with baseline.

**P < 0.01 compared with baseline.

**Table 3:** Clinical responses at weeks 4, 8, 12, and 24.

End point	Week 4	Week 8	Week 12	Week 24
*End points reported by the physician *				
PASI 50 response				
number of patients (%)	2 (20)	3 (30)	5 (50)	3 (30)
PASI 75 response				
number of patients (%)	0 (0)	0 (0)	2 (20)	0 (0)
Mean PASI (SD)	5.2 (3.3)	3.2 (1.2)	3.2 (1.3)*	3.8 (1.6)*
“Most clear” or “mild” status				
Number of patients (%)	5 (50)	7 (70)	8 (80)	7 (70)

*End points reported by the patient *				
Percentage improvement in Dermatology Life Quality index (SD)	34.1 (30.1)	46.9 (34.5)	62.5 (26.0)**	40.4 (33.5)*
Overall treatment satisfaction score (SD)	8.00 (2.4)**	9.2 (1.3)**	9.4 (1.1)**	8.6 (1.2)**
Rash satisfaction score (SD)	6.1 (3.1)*	8.6 (1.4)**	9.3 (0.8)**	8.8 (1.4)**
“Most clear” or “mild” status				
Number of patients (%)	4 (40)	7 (70)	9 (90)	6 (60)

*P < 0.05 compared with baseline.

**P < 0.01 compared with baseline.

**Table 4:** Serum variables for the study population at baseline and weeks 12 and 24.

	Baseline	Week 12	Week 24
Mean hemoglobin, mg/dL (SD)	14.5 (1.0)	14.2 (0.9)	14.3 (1.3)
AST, U/L (SD)	34.6 (12.5)	23.3 (7.7)*	22.1 (4.8)**
ALT, U/L (SD)	57.1 (26.2)	40.0 (6.5)*	38.4 (7.2)
Creatinine, mg/dL (SD)	1.01 (0.2)	0.8 (0.2)*	0.9 (0.1)*
Fasting blood sugar, mg/dL (SD)	116.2 (33.5)	104.8 (18.1)	108.7 (26.8)
HbA1c, % (SD)	5.9 (2.5)	6.1 (0.7)	5.8 (0.6)
Triglyceride, mg/dL (SD)	157.9 (83.2)	100.2 (45.3)**	93.5 (34.5)*
Total cholesterol, mg/dL (SD)	193.9 (34.7)	175.4 (29.6)	205.4 (33.5)
HDL cholesterol, mg/dL (SD)	43.5 (8.9)	44.9 (7.0)	49.3 (10.6)*
LDL cholesterol, mg/dL (SD)	124.0 (29.4)	110.1 (26.3)*	136.5 (34.9)
LDL : HDL cholesterol ratio (SD)	2.9 (0.8)	2.4 (0.6)*	2.8 (0.8)
hs-CRP, mg/dL (SD)	5.9 (4.2)	5.6 (5.9)	5.6 (6.4)
ESR, mm/hr (SD)	20.9 (10.6)	19.0 (8.8)	16.3 (9.7)

*P < 0.05 compared with baseline.

**P < 0.01 compared with baseline.
